# Reconfigurable Mechanochromic Patterns into Chameleon-Inspired Photonic Papers

**DOI:** 10.34133/2022/9838071

**Published:** 2022-07-19

**Authors:** Dongpeng Yang, Yang Hu, Dekun Ma, Jianping Ge, Shaoming Huang

**Affiliations:** ^1^School of Materials and Energy, Guangzhou Key Laboratory of Low-Dimensional Materials and Energy Storage Devices, Guangdong University of Technology, Guangzhou 510006, China; ^2^Zhejiang Key Laboratory of Alternative Technologies for Fine Chemicals Process, Shaoxing University, Shaoxing 312000, China; ^3^School of Chemistry and Molecular Engineering Shanghai Key Laboratory of Green Chemistry and Chemical Processes, East China Normal University, Shanghai 200062, China

## Abstract

Photonic crystal (PC) patterns have shown wide applications in optical devices, information encryption, anticounterfeiting, etc. Unfortunately, it is still a great challenge to reconfigure the PC patterns once fabricated. Herein, a new strategy is presented to reconfigure self-recordable PC patterns by printing local patterns into the chameleon-inspired PC papers using the phase change material (PCM) as ink and then erasing the patterns in ethanol. Multicolor and high-resolution (25 and 75 *μ*m for dot and lines, respectively) patterns can be efficiently and repeatedly reconfigured. In addition, the photonic patterns based on the PC paper and PCM combinations are gifted with mechanochromic characteristics and can show programmable and reversible color change under pressure. The high melting point of the ink, nonclosely packed structures of the PC paper, and the similar solubility parameter of PC paper, PCM, and ethanol are the keys for all these characteristics. This work offers a simple, flexible, efficient way to reconfigure PC patterns with mechanochromic properties and could open up exciting applications for novel hand-operation-based anticounterfeiting and optical devices.

## 1. Introduction

Photonic crystal (PC) patterns [1, 2] have attracted numerous interests due to their wide applications in displays [3–9], sensing [10–18], pigments [19–22], devices [23–30], anticounterfeiting [31–40], photocatalysis [41], optical coatings [42, 43], and solar cells [44]. The conventional PC patterns can be fabricated by ink-jet printing [45–50] using the colloidal solution as inks and selective altering of photonic structures by compression [51], particle aggregations [52], magnetic field [53], and electric field [54, 55] with the assistance of photopolymerization and templates. Although PC patterns can be prepared in a low-cost and efficient way, a fundamental drawback is that most patterns are permanent and cannot be reconfigured because the structures of the patterned regions are chemically and irreversibly fixed.

To reconfigure the PC patterns, the following criteria should be met: (1) the patterns into PC papers should possess recordable and self-maintained characteristics of structural color at normal conditions; (2) multicolor and high-resolution patterns can be erased at specific stimuli to recover the PC papers; (3) there is full repeatability of reconfiguration; and (4) for practical operation, the applied stimulus should be simple, convenient, and efficient excluding complicated procedures and equipment.

Recently, considerable methods and strategies have been developed to reconfigure PC patterns based on the cholesteric liquid crystals and shape memory polymers and electro-responsive polymers. PC patterns can be reconfigured on the cholesteric liquid crystal polymer network using liquid crystal as the ink and tetramethylene oxide (THF) as the eraser. The printed patterns were stable, and the color of the patterned region can be tuned by the amount of the ink [56–59]. However, the usage of THF may restrict their practical applications. The reversible destruction of shape memory PC substrates [60–68] with inverse opal structures is one of the most widely used strategies to reconfigure PC patterns. Generally, a pattern can be created by applying pressures onto PC substrates and then erased when exposed to external stimuli, such as solvent vapors and heating. However, this strategy only can achieve transparent patterns rather than multicolor patterns, which will limit their utility. Additionally, PC patterns can be reconfigured through water-writable and electro-erasable poly(3,4-ethylenedioxythiophene)-based inverse opal structures [69] or by writing and then erasing the patterns by the electronic field on the nanocomposite integrated with Fe_3_O_4_@C-based PCs and bistable electroactive polymer [70]. Nevertheless, the requirements of delicate fabrications, extra electric fields, and equipment will restrict their practical applications. Moreover, it is difficult for the aforementioned approaches to reconfigure patterns with high resolution.

**Figure 1:**
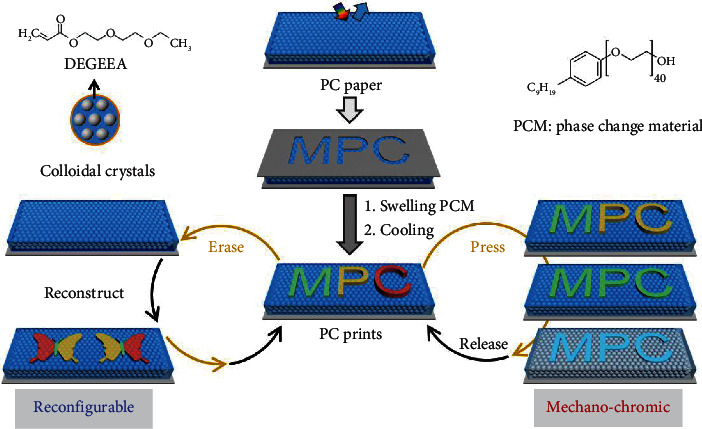
Schematic illustration of the reconfiguration of multicolor and high-resolution PC patterns with mechanochromic characteristics.

In this paper, we present a new method fulfilling all the abovementioned requirements to repeatedly reconfigure PC patterns based on the rational design and combinations of chameleon-inspired swellable PC papers with nonclosely packed structures and phase change material IGEPAL CO-890 (PCM, CO890). The reconfigurable processes include the preparation of self-recordable and stable patterns by region-selective swelling of PC papers using PCM as the ink and then erasing the patterns by removing PCM in ethanol (Figure 1). Compared with the previous methods, the reconfigurable strategy in this work is simple, facile, and effective. Full-color and high-resolution patterns can be reconfigured repeatedly in a green and low-cost way. The high fraction of swellable polymer of the PC paper, the high phase transition temperature of PCM, and the close solubility parameter between the PC paper and PCM are the keys to the successful reconfigurations of patterns. In addition, the combination of the PC paper and PCM endows the patterns with color-dependent mechanochromic performances under pressure. The pattern can show programmable and reversible changes of the colors in response to pressures. This work offers a new way to reconfigure PC patterns with mechanochromic characteristics and will facilitate their practical applications in hand-operation-based green printing, optical devices, and anticounterfeiting.

## 2. Results and Discussion

### 2.1. Characterization of Chameleon-Inspired Swellable & Mechanochromic SiO_2_-DEGEEA PC Papers

**Figure 2:**
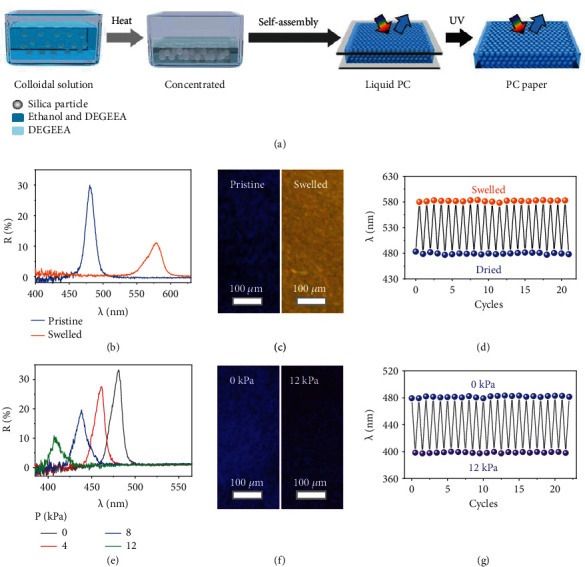
Swellable and mechanochromic PC paper. (a) Schematic illustration of the fabrication of PC paper. (b) Reflection spectra and (c) digital photos of the PC paper at the pristine state and after being swelled in propanol. (d) The switch of the reflection wavelength of the PC paper at swelled and dried states. (e) Reflection spectra and (f) microscope images of the PC paper under different pressures. (g) The switch of the reflection wavelength of the PC paper with cycles of pressing-releasing.

The chameleon-inspired solvent and pressure-responsive PC paper were firstly prepared by the self-assembly of the silica particles into the di(ethylene glycol) ethyl ether acrylate (DEGEEA) with the assistance of photopolymerization (Figure 2(a)). The volume fraction of the silica particles and DEGEEA is designed to be 40% and 60%, respectively, so that the PC paper can possess a structure and mechanochromic characteristic similar to those of chameleons and thus good swellable properties for printing full-color patterns. As shown in Figure 2(b) and Figure S1(a), at the reflection mode, the as-prepared PC paper shows brilliant blue color with a reflection wavelength located at around 480 nm. The narrow full width at half maximum (FWHM) (17 nm) of the reflection peak indicates the long-range ordered structure of the PC paper. At the transmission mode, the PC paper is optically transparent with transparency reaching nearly 100% across all the visible range out of the photonic bandgap since the efficiency of incoherent scattering of light by the ordered structures is greatly suppressed with the small contrast of the refractive index between the silica particles (1.46) and DEGEEA (1.47). The PC paper possesses nonclosely packed structures since the volume fraction of silica particles is far below that (74%) of closely packed structures. The surface-to-surface (*D*_*s*−*s*_) distance between neighboring particles can be calculated by Bragg's law (equation (1)) and equation (2) as follows:

(1) $$\begin{array}{c} m\lambda =1.633D_{\text{id}}{\left(n^{2}-\sin^{2}\theta \right)}^{1/2}, \end{array}$$

(2) $$\begin{array}{c} n^{2}={n_{\text{s}}}^{2}f_{\text{s}}+{n_{\text{d}}}^{2}f_{\text{d}}, \end{array}$$

(3) $$\begin{array}{c} D_{\text{s}‐\text{s}}=D_{\text{id}}-D_{\text{s}}, \end{array}$$

where *m* and *λ* are the diffraction order and reflection wavelength, respectively. *D*_id_ is the interparticle distance of neighboring particles. *θ* is the angle between the normal and reflective light. The *n*, *n*_s_, and *n*_d_ are the refractive index of the PC paper, silica particles, and DEGEEA, respectively. *f*_s_ and *f*_d_ are the volume fraction of silica particles and DEGEEA, respectively. Here, *D*_s−s_ is calculated to be 33.6 nm, implying the nonclose packing of the silica particles in DEGEEA. The ordered structures and nonclosely packed structures of the PC paper can be confirmed by its scanning electron microscope (SEM) (Figure S1(b)) image. Under SEM, the silica particles are nonclosely packed to each other and *D*_s−s_ is measured to be 38.2 nm, in good agreement with the result calculated from the reflection spectrum. The 2D and 3D angle-resolved reflection spectra (Figure S1(c)) of the PC paper show that its reflection wavelength blue shifts from 480 to 393 nm and the corresponding color changes from blue to purple (Figure S1(d)) when the incident and detection angle decrease from 0 to 60° simultaneously, further verifying the long-range ordered structures of the PC paper. These results firmly demonstrated that the PC paper possesses highly ordered and nonclosely packed structures similar to those of chameleon skins [71–74].

The reflection wavelength and structural color of PC paper can be precisely controlled by altering the size of silica particles. For example, PC papers with reflection wavelengths located at 532, 581, and 630 nm and the corresponding green, yellow, and red colors (Figure S2) can be prepared when silica particles with sizes of 180, 192, and 213 nm are used, respectively. These PC papers also have long-ranged structures and nonclosely packed structures (Figure S3). In the following part, the blue PC paper with a particle size of 170 nm, *f*_s_ of 40%, and reflection wavelength located at 480 nm is used to demonstrate our concept unless otherwise specified.

The highly ordered structures of the PC paper originate from the strong electrostatic forces between silica particles in DEGEEA. Generally, it is hard for the silica particles to be charged in less polar DEGEEA due to the negligible dissolution of counterions in DEGEEA [75]. The counterions dissolved in ethanol usually refer to the H^+^ released from the surface of silica particles (silica-OH → silica-O^−^ + H^+^). Fortunately, a trace amount of ethanol (~6.0%) exists in the liquid PC after evaporation, which acts as the charge controller by dissolving the counterions, leading to the significant reduction of the energy barrier of charge separation and long-range electrostatic repulsions between particles [76–79]. The threshold of the crystallization of the silica particles in DEGEEA is 8% (Figure S4), and the corresponding *D*_s−s_ is calculated to be 152.3 nm, ruling out the Van der Waals force as the driving force since such a large interparticle distance already exceeds the effective distance of Van der Waals force [80]. The silica particles as the building blocks self-assemble into the ordered structure under the electrostatic force and improve the mechanical property of the PC paper. The particle-free matrix polymer of DEGEEA is easily fragile and shows a small strain [81]. The mechanical property of the PC paper is shown in Figure S5, with the maximal strain of 480% and the maximal stress of 720 kPa, which is better than the particle-free DEGEEA due to the physical crosslinking of silica particles.

The PC paper exhibits pronounced swellable properties due to the high-volume fraction of DEGEEA. As shown in Figures 2(b) and 2(c), after immersing in propanol for ~3 min, the reflection signal of the PC paper shifts from 480 to 578 nm and the color changes from blue to yellow accordingly. The redshift of the reflection wavelength can be attributed to the increase of lattice distances of the PC paper by swelling. After drying, the PC paper recovers to the pristine state and the switch of the reflection signal between the swelled and dried states is fully reversible (Figure 2(d)).

Except for the swellable characteristics, the PC paper shows outstanding mechanochromic properties due to the nonclosely packed structures. As presented in Figures 2(e) and 2(f), the reflection wavelength of PC paper blue shifts from 480 to 407 nm and the corresponding color turns from blue to violet as the pressure increases from 0 to 12 kPa accordingly. The mechanochromic sensitivity of the PC paper is ~6.1 nm/kPa, one of the best values of opal-based PCs [7, 82, 83]. The blue shift of reflection peak position under pressures can be attributed to the decrease of lattice distance of (111) planes. As demonstrated in Figure S6, the lattice distance of the (111) plane of the PC paper decreases from 140.3 to 132.1 nm as the pressure increases from 0 to 12 kPa. According to Bragg's law, the decrease of the lattice distance will cause the blue shift of reflection wavelength. In addition, it only takes about 30 ms (Figure S7) for the PC paper to finish the switching of the reflection wavelengths under pressures, indicating its fast responsive speed (2.4 nm/ms). After releasing the pressure, the PC paper can quickly recover to the pristine state and the switch of the reflection signals between 0 and 12 kPa is fully reversible (Figure 2(g)).

### 2.2. Multicolor and High-Resolution PC Patterns

**Figure 3:**
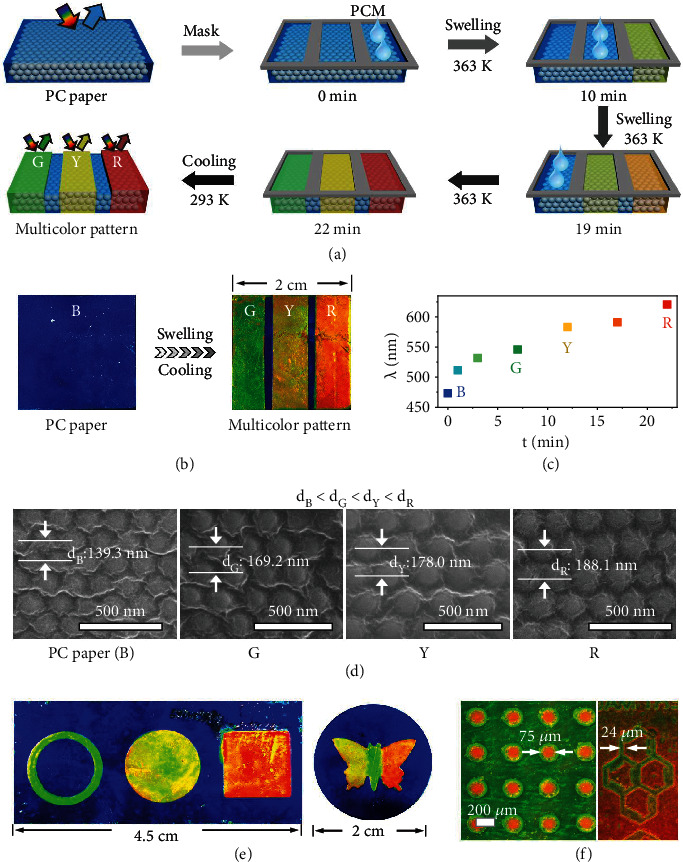
Fabrication of multicolor and high-resolution PC patterns by controlling the swelling time. (a) Schematic illustration of the fabrication of PC patterns with multicolor patterns. (b) Digital photos of PC paper and as-prepared multicolor pattern. (c) Reflection spectra of the B, G, Y, and R points. (c) Reflection wavelength as a function of the swelling time. (d) Cross-sectional SEM images of patterned regions with different colors. From left to right: B, G, Y, and R. (e, f) Digital photos of the multicolor, large-area, and high-resolution patterns.

The good swelling ability of the PC paper in propanol inspires us to fabricate PC patterns with multicolor and self-recordable patterns through the localized swelling of the PC paper by the PCM (CO890) as the ink with the help of the masks. As shown in Figures 3(a) and 3(b), the pristine PC paper shows a bright blue color. The mask with three hollow rectangle patterns was covered onto the PC paper to achieve three regions with the same areas. Excessive amounts of preheated liquid PCM were cast sequentially on these bare regions to initiate the swelling at 363 K. Due to the good swelling properties, the PCM could be absorbed into the polymer network of the PC paper, leading to the increase of the lattice distance and redshift of the reflection wavelength of the patterned regions. The swelling can be stopped immediately once the swelled PC paper was cooled down to room temperature (RT) and the multicolor patterns were obtained after removing the mask. Thus, it is feasible to adjust the structural colors and reflection peak positions of the patterns by precisely controlling the swelling time. As demonstrated in Figure 3(b), the patterns with brilliant green, yellow, and red colors and corresponding reflection wavelengths located at 530, 583, and 618 nm, respectively, (Figure 3(c) and Figure S8) can be achieved when the swelling time is set to be 3, 12, and 22 min, respectively. Apparently, a long swelling time will cause a large mass fraction of the CO890 (*m*_CO890_%: from 15.0 to 24.7% wt) and thus a large expansion of the lattice distance (Figure 3(d) and Figure S9, from 139.3 to 188.1 nm) and shift of the reflection wavelength (Δ*λ*: from 50 to 138 nm). According to Bragg's law, the increase of lattice distance will cause the redshift of the reflection wavelength. These results prove that the reflection wavelength of patterns can be precisely controlled through altering the swelling times. In addition to the swelling time, other parameters including the thickness of the PC paper and the *f*_d_ also have significant influences on the optical properties of the patterns. The detailed results and discussion can be found in supporting information (Figure S10). Overall, a thin PC paper and high *f*_d_ will be favorable for swelling.

Multicolor and high-resolution patterns can be easily fabricated by altering the masks and the swelling time (Figures 3(e) and 3(f)) based on the region selective swelling strategy. As shown in Figure S11(a), the delicate mask with circular array patterns was covered on the green PC paper and excessive amounts of preheated liquid PCM were cast on these bare regions to swell at 363 K for 2 min. Then, the yellow patterns with a reflection wavelength of 578 nm were generated after removing the mask (Figure S11(b)). Similarly, the red patterns with a reflection wavelength of 620 nm can be obtained by swelling the green PC paper for 10 min (Figure S11(c)). The rigid lines, boundaries, and other details of the macro-/micropatterns are legible, indicating the high-resolution characteristic. The width of the line and the diameter of the point are measured to be 24 and 75 *μ*m, respectively.

The successful fabrication of multicolor patterns can be attributed to the controllable swelling of the PCM into DEGEEA of the PC paper. Here, the solubility parameter (*δ*), an important parameter that can characterize the swelling ability of the polymer in solvents, is used to gain a deep understanding of the interactions between the DEGEEA and the PCM. In general, a small contrast of the solubility parameter (Δ*δ*) between the polymer and the solvent will facilitate the swelling. Otherwise, the polymer cannot be swelled by the solvents. The *δ* of the polymer and solvent can be calculated by equation (4), where *F*_i_ and *M*_0_ are the molar attraction constant and the molecular weight of the structural unit, respectively. The *δ* of the DEGEEA and CO890 is calculated to be 17.8 and 14.3 (J/cm^3^)^1/2^, respectively, and the Δ*δ* is small that should be responsible for the good swelling. To demonstrate this hypothesis, water (*δ*_water_ = 47.3 (J/cm^3^)^1/2^) and ethanol (*δ*_ethanol_ = 26.0 (J/cm^3^)^1/2^) with different solubility parameters are used to test the swelling capability of the PC paper. As expected, a new reflection peak located at 823 nm quickly appears once the PC paper is immersed in ethanol, proving that the PC paper can be swelled by ethanol (Figure S12) due to the small Δ*δ*. In striking contrast, the swelling of the PC paper in water is negligible, as evidenced by its nearly unchanged reflection wavelengths (Figure S13) because of the large Δ*δ*. These results manifest that the small Δ*δ* between DEGEEA and PCM is the key to the successful fabrication of the multicolor patterns. Other PCMs including Brij S2, Brij S10, and Brij S100 cannot be used as inks to reconfigure PC patterns due to the unfavorable solidification of the PCMs on the PC paper or the large Δ*δ* between the ink and DEGEEA (Figure S14a-b).

(4) $$\begin{array}{c} \delta =\rho \sum \frac{F_{\text{i}}}{M_{0}}. \end{array}$$

### 2.3. Recordable and Reconfigurable PC Patterns

The as-prepared multicolor patterns show long-term stability at the normal circumstance, suggesting that the information was recordable. The key to the recordable and stable patterns is to select the PCM with a phase transition temperature (*T*_m_) above the RT. For comparison, CO720 with similar structures to that of CO890 but *T*_m_ < RT is used as the ink to fabricate the patterns (Figure S15(a)). The blue-colored regions represent the backgrounds, while the regions with red colors are the regions swelled either by CO720 or CO890, respectively. For the pattern of CO720 (Figure S15(b)), the ink diffuses from the red to the blue-colored regions (white arrow) and the diffusion distance increases from 0 to 280.4 *μ*m when the times increase from 0 to 24 h. This means that the pattern cannot be recorded owing to the liquid state of CO720 at RT. On the contrary, for patterns of CO890 (*T*_m_ > RT), no diffusion can be observed at the microscale and the details are well retained for at least 24 h due to the solid state of CO890 at RT. As shown in Figure S15(c), the reflection wavelengths of the patterns are constant over 2 months, demonstrating the long-term stability. The PC patterns with half regions swelled by CO890 (*T*_m_ = 46°C) were placed under 30, 50, 70, and 90°C for an hour (Figure S16(a)). At 30°C, the pattern is stable and no diffusion of the ink can be observed due to *T*_m_ > 30°C. In contrast, at other temperatures (50–90°C), the ink transforms from the solid to the liquid state, leading to the diffusion of the ink over time, as evidenced by the green-colored regions caused by the ink diffusion. The diffusion speed of the ink (*k*) is proportional to the temperature, suggesting that a higher temperature will cause a blurry pattern in a shorter time (Figure S16(b)). These results demonstrate that the pattern is stable only when the temperature is below the *T*_m_ of CO890. The *T*_m_ of the CO890 is much higher than the room temperature so that the photonic pattern can be well retained under normal conditions.

**Figure 4:**
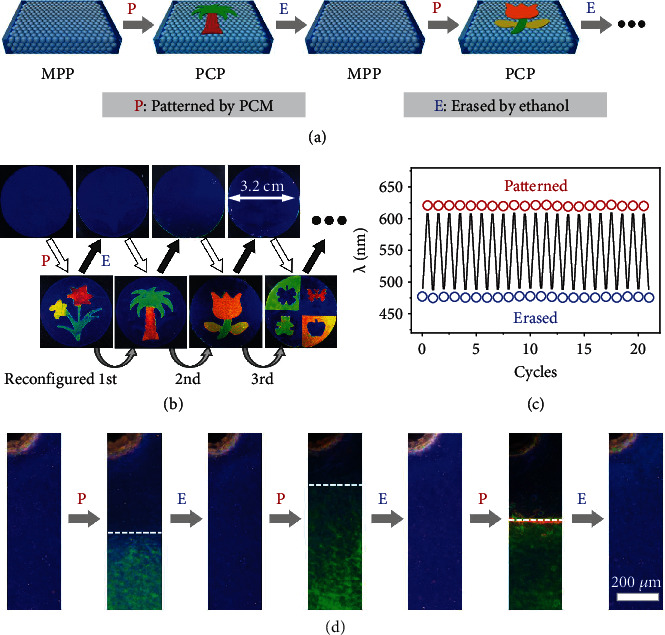
Reconfiguration of PC patterns. (a) Schematic illustration of the reconfiguration process. (b) Digital photos of PC paper and patterns of each reconfiguration. (c) Switch of reflection spectra of the PC patterns as a function of reconfigurable cycles. (d) Microscope images of PC paper repeatedly patterned and erased by the PCM.

The patterns can be reconfigured by erasing the premade pattern and then reconstructing a new pattern on the PC paper with similar procedures (Figure 4(a)). The pattern can be erased by extraction of the PCM from the PC paper in ethanol because PCM is not chemically but physically immobilized into the networks of DEGEEA. Here, ethanol has two functions. First, the ethanol can dissolve the PCM, allowing the extraction process. Second, ethanol can swell the PC paper quickly, resulting in the free infiltration of ethanol into the network and accelerating the extraction process. The pattern disappears once CO890 is fully extracted from the PC paper by ethanol, and the PC paper with a blue color can be recycled after drying. It takes about 10 min to erase the pattern at RT, and the time can be significantly decreased to 2–3 min at higher temperatures, such as 333 K. New patterns can be printed onto the recovered PC paper through the region-selective swelling strategy. Figure 4(b) demonstrates that multicolor patterns can be repeatedly reconfigured on the same PC paper. The erased regions show similar color to the background, indicating the successful removal of PCM from the PC paper. The clear boundaries of all the patterns suggest that each reconfiguration process has a neglectable influence on the others. It is worth noting that the patterning-erasing process is fully reversible and can work effectively more than 20 times (Figure 4(c)), as demonstrated by the periodic switch of the reflection wavelength upon the patterning-erasing process.

Erasing by ethanol was thorough, and the reconfigured pattern was not affected by the erase pattern, which can be strictly examined by the optical microscope. As shown in Figure 4(d), the pristine PC paper shows a blue color. After being partially patterned by PCM, the PC paper shows obvious blue-green color contrast, in which the green region represents the swelled part. The pattern was erased by ethanol and dried, and the whole PC paper was recovered to the pristine state, and no trials of other colors can be observed, indicating the complete removal of PCM at the microscale. With additional cycles of reconfiguration, the recycled PC paper still shows a blue color, which means that the PCM would not be accumulated or retained into the network of the PC paper. This characteristic inspires us to reconfigure patterns at microscales. As illustrated in Figure S17, high-resolution patterns with green-red color contrast can be repeatedly reconfigured with high efficiency. The patterns of each cycle exhibit brilliant structural colors, and the details of the patterns can be clearly observed. To the best of our knowledge, this is the first time that micropatterns can be repeatedly reconfigured.

Compared to the previous works, the reconfigurable approach in this work has the following advantages: (1) the reconfigurable process is considerably simple, efficient, and convenient because no special equipment or hard conditions are required during the whole reconfigurable process; (2) the inks and erasers can be recycled through selective evaporation of ethanol from their mixtures based on the large contrast of their boiling points, facilitating the economic and green fabrications; and (3) multicolor and high-resolution patterns can be easily reconfigured at a large scale. These characteristics will make the reconfigurable process more controllable, flexible, convenient, and scalable.

### 2.4. Mechanochromic Patterns for Anticounterfeiting Applications

**Figure 5:**
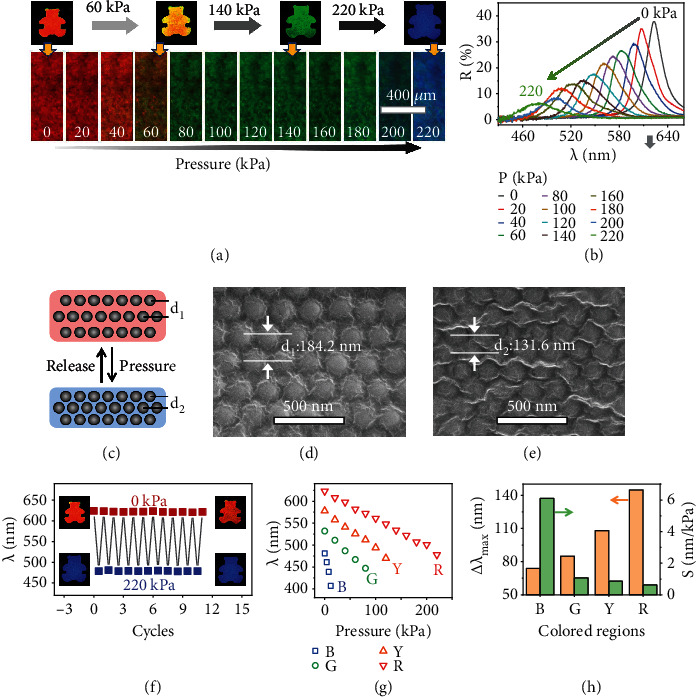
PC patterns showing color-changeable patterns under pressures. (a) Digital photos and microscope images and (b) reflection spectra of the P-623 under different pressures. (c) Schematic illustration and (d, e) cross-sectional SEM images of the patterned region with red color in the (d) absence and (e) presence of pressure (220 kPa). (f) Switch of the reflection wavelength of the P-623 with red color in the absence and presence of pressure (220 kPa). (g) Reflection wavelength of the PC paper and P-532(G)/578(Y)/623(R) as a function of pressure. (h) The Δ*λ*_max_ and *S* depend on the reflection wavelengths and structural colors of P-532(G)/578(Y)/623(R).

The combinations of the PC paper and PCM endow the patterns with a broad tuning range of the reflection wavelength (Δ*λ*_max_) in response to pressures. Here, four samples including the one pristine PC paper and three PC patterns with different reflection wavelengths located at 532, 578, and 623 nm are prepared independently and named P-532, P-578, and P-623, respectively. Here, the P-623 (Figure 5(a)) is used as the typical example for further investigations. With the pressure gradually increased from 0 to 220 kPa, the appearance of the P-623 turns from brilliant red to blue instantly (<1 s) and its reflection wavelength (Figure 5(b)) changes from 623 to 478 nm accordingly. The Δ*λ*_max_ of P-623 is 145 nm, which is almost 2 times larger than that of the PC paper. The broad-spectrum responsiveness of P-623 originates from its large lattice distance which leaves more space for compression. The blueshift of the reflection signal can be attributed to the shrinkage of the lattice distance under pressures (Figure 5(c)). Under SEM (Figures 5(d) and 5(e)), the lattice distance of the (111) plane decreases from 184.2 to 131.6 nm when the pressure increases from 0 to 220 kPa, proving the shrinkage of the lattice distance under pressure. According to Bragg's law, the decrease of lattice distance will lead to the decrease of the reflection wavelength and the variation of the structural color of the pattern accordingly. The reflectance decreases, and the full width at half-maximum (FWHM) broadens as the pressure increases, suggesting that the order degree of the pattern is reduced by external pressure. After releasing the pressure, the color of the pattern quickly recovers back to the pristine state and the press-release switch is fully reversible, as substantiated by the periodic change of the reflection signal at different states (Figure 5(f)). The PC pattern with a half region swelled by CO890 was pressed 20 times. As illustrated in Figure S18, the border of the PC pattern is clear after 20 times of being squeezed, implying that the resolution of PC patterns is independent of the processes of the pressing-releasing.

The abovementioned results suggest that the Δ*λ*_max_ and mechanochromic sensitivity (*S*) of patterns depend on their initial structural colors and reflection wavelengths after swelling. Figure 5(g) and Figure S19 show that the reflection wavelength of the PC paper and P-532/578/623 blue shift as the pressure increases. The Δ*λ*_max_ (Figure 5(h)) increases in combination with the reflection wavelength of the pattern. The Δ*λ*_max_ of P-532/578/623 is 85/108/145 nm, respectively. In striking contrast, the *S* of the pattern decreases dramatically when the reflection peak position increases. The *S* of the PC paper is 6.2 nm/kPa, while those of the P-532/578/623 are 1.1/0.9/0.7 nm/kPa, respectively. The decrease of the *S* can be explained by the fact that the patterned regions with solid PCM are harder than those of the PC paper, which needs a larger pressure to achieve the same shift of the reflection wavelength. Therefore, the *S* of the patterned region is inversely proportional to the value of reflection wavelength. These results demonstrate that the Δ*λ*_max_ and *S* can be controlled by altering the reflection wavelength of the pattern.

**Figure 6:**
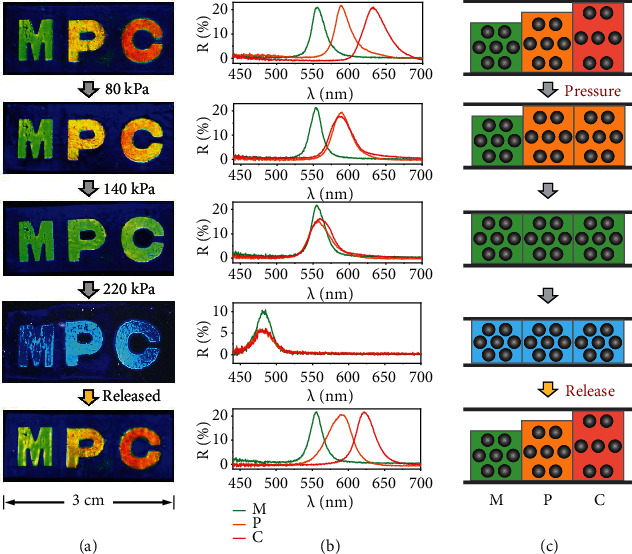
Pressure-based anticounterfeiting application. (a) Digital photos, (b) reflection spectra, and (c) schematic illustration of the variation of the multicolored “MPC” under pressure.

A new mode of pressure-based anticounterfeiting is developed based on the diverse Δ*λ*_max_ and thicknesses of the patterns. The patterns with diverse colors have different thicknesses, which will lead to the programmable change of colors under pressure. Here, multicolored “MPC” patterns (Figure 6(a)) with the blue background were prepared and used for the proof of the concept to show its potential in mechanochromic anticounterfeiting applications. The “M,” “P,” and “C” show brilliant green, yellow, and red colors with corresponding reflection wavelengths located at 555, 588, and 633 nm (Figure 6(b)), respectively. As discussed, the lattice distance and thickness of the pattern are increased after swelling, which can be approximately determined by its reflection wavelength and corresponding colors. For the “MPC” pattern, the thickness (*T*) of the patterns and background follows (Figure 6(c) and Figure S20): *T*_B_ < *T*_M_ < *T*_P_ < *T*_C_, where B represents the blue background. When the pressure (80 kPa) is applied to the pattern, the “C” will be firstly compressed since it has the largest thickness. The compression stress will cause the decrease of the lattice distance, resulting in the blue shift of the reflection and color change of the “C” from red to yellow while other patterns keep unchanged, implying the programmable color change of patterns under pressure. The similar colors and reflection wavelengths of “C” and “P” indicate that these two patterns have similar thicknesses and lattice distances. Similarly, when pressure is increased to ~140 kPa, the patterns of the “PC” can respond to the pressure by altering their colors from yellow to green. Thus, the patterns of “MPC” showing similar green colors and reflection wavelengths are obtained. Further increasing the pressure (220 kPa) will induce the uniform color change of all these patterns from green to blue. Therefore, the “MPC” patterns can show programmable changes of their colors under pressure. After releasing the pressure, the colors of patterns can quickly recover to the pristine state. The programmed change of the colors of the patterns under pressure may advance their applications in anticounterfeiting and information storage. It should be mentioned that the repetitive pressing of the pattern has a neglectable effect on the reconfiguring property. For example, the “MPC” patterns can be repeatedly reconfigured (Figure S21) after experiencing 5 cycles of the pressed-released process.

## 3. Conclusion

In summary, we developed a facile and green method to reconfigure PC patterns through printing the patterns based on the region-selective swelling of the bioinspired PC paper with the PCM as ink and then erasing the patterns by extraction of the PCM from the PC paper in ethanol. The similar solubility parameters among PCM, PC paper, and ethanol drive the swelling and erasing process. A long swelling time, a thin PC paper, and a high value of *f*_d_ are favorable for the swelling and a large reflection wavelength. Multicolor and high-resolution patterns can be repeatedly reconfigured, which exhibit long-term stability and recordable properties at normal conditions. In addition, the patterns show different mechanochromic properties depending on their initial reflection wavelengths and structural colors after swelling. Based on these unique characteristics, the as-fabricated patterns were used for a new pressure-based anticounterfeiting application with the programmable change of the colors of the patterns under pressure. The simple and efficient reconfiguration of PC patterns together with mechanochromic characteristics will facilitate their practical applications in the fields of green printing, visualized sensors, information encryption, and high-level anticounterfeiting.

## 4. Materials and Methods

### 4.1. Materials

Tetraethyl orthosilicate (TEOS, 98%), ethanol (EtOH, 99%), and aqueous ammonia (28%) were purchased from Sinopharm Chemical Reagent Co. Ltd. Di(ethylene glycol) ethyl ether acrylate (DEGEEA, 90%), Brij S2 (Mn: 358.6), Brij S10 (Mn: 711), Brij S100 (Mn: 4670), IGEPAL CO720 (Mn: 749), CO890 (Mn: 1982), and 2-hydroxy-2-methylpropiophenone (photoinitiator, 96%) were obtained from Sigma-Aldrich. All the chemicals were used as received without further purifications.

### 4.2. Preparation of the Chameleon-Inspired Swellable PC Paper

The PC chameleon-inspired papers are prepared by the self-assembly of silica particles into DEGEEA according to our previously reported work [81]. Typically, Stöber silica particles (0.04 mL) with the size of 170 nm were dispersed in the mixture of ethanol (1.1 mL) and DEGEEA (0.06 mL) containing a 5% photoinitiator with the assistance of sonication. Afterward, the mixed solution was heated at 373 K for 1 h in an oven and then a liquid precursor can be obtained. The precursor solution (0.03 mL) was sandwiched between two glasses with an interval of 0.09 mm, which was then exposed to the UV light (365 nm, 4.8 mW·cm^−2^) for 3 min to fabricate the blue PC paper. The distance between the UV light source and sample is 15 cm. The volume fraction of silica particles and DEGEEA is 40% and 60%, respectively. The reflection wavelength and structural color of the PC paper can be well controlled through altering the size of particles. For example, PC papers with reflection wavelengths located at 532, 581, and 630 nm and corresponding green, yellow, and red colors are prepared when particles with the sizes of 180, 192, and 213 nm are used, respectively.

### 4.3. Printing of PC Patterns

Firstly, the blue PC paper was covered by a mask with a designed hollow pattern. Secondly, the preheated liquid PCM (CO890) was cast on the pattern regions of the PC paper. Thirdly, the PC paper was heated at 363 K in order to accelerate the swelling process. The color and the reflection wavelength of the pattern can be controlled by adjusting the swelling times. For example, the pattern with green, yellow, and red colors and reflection peak positions located at 530, 578, and 620 nm, respectively, can be obtained when the time is 3, 12, and 22 min, respectively. Lastly, a multicolor pattern can be achieved as the film was cooled down to room temperature, and then, remove the mask.

### 4.4. Reconfigure the PC Patterns

The pattern can be erased after immersing in excessive ethanol (20 mL) for 10 min to extract CO890 out. Afterward, the PC paper swelled by ethanol was dried in the oven (typically: 363 K for 2 min), which shows similar structural color to that of the pristine one. Then, a new pattern can be printed on the dried PC paper with similar fabrication procedures. Multicolor and high-resolution patterns can be reconfigured repeatedly through combing the printing and erasing processes.

### 4.5. Characterization

The structures of PC papers were investigated by the HITACHI SEM-SU8010. As illustrated in Figure S22, for most cases, the reflection spectra of PC papers are obtained by mode 1 with a collection angle fixed at 0°. The reflectance spectra were measured by a NOVA spectrometer (Hamamatsu, S7031). Angle-resolved spectra are collected by mode 2 with reflection angle changes from 0 to 60°, using the angle-resolved spectrum system (R1, Ideaoptics, China) equipped with a highly sensitive spectrometer (NOVA, Ideaoptics, China). The optical microscope images and microscopic reflectance spectra were obtained on an Olympus BXFM reflection-type microscope operated in a darkfield mode. The mechanochromic tests of the PC paper and patterns are illustrated in Figure S23. The PC paper or pattern was sandwiched between the two glass slides, and the optical probe was fixed on the top of the PC paper or patterns to record the reflection spectra under pressure. Then, objects with desired weight were added on the glasses and the desired pressure (*P*) can be calculated by *P* = *mg*/*S*, where *m* is the mass of the objects and *g* is the acceleration of gravity. *S* is the surface area of one side of the PC paper or PC pattern. Therefore, one can change the pressure loaded on the PC paper or PC pattern by altering the mass of the objects.

## Data Availability

The data that support the findings of this study are available from the corresponding author upon request.

## Supplementary Materials

Supplementary Materials Supplementary Materials Figure S1: schematic illustration of two modes for the collection of the reflection spectra of the PC paper or PC patterns. Figure S2: schematic illustration of the details of testing the mechanochromic properties of the PC paper or PC patterns. Figure S3: optical properties and microstructure characterization of the PC paper. Figure S4: reflection spectra of the PC papers prepared with different volume fractions of silica particles. Figure S5: reflection spectra and corresponding digital photos of the PC papers self-assembled from silica particles with different sizes. Figure S6: SEM images of PC papers self-assembled by silica particles of different sizes. Figure S7: SEM images of the PC paper under (a) 0 and (b) 12 kPa. Figure S8: 3D reflection spectra of the PC paper with the pressure increased from 0 to 12 kPa. Figure S9: reflection spectra of the B, G, Y, and R points. Figure S10: mPC and mCO890 and the mCO890% and Δ*λ* as a function of the thickness and *f*_d_ of the PC paper. Figure S11: reflection spectra of the PC paper at a pristine state and in ethanol. Figure S12: reflection spectra of the PC paper at a pristine state and in water. Figure S13: CO720 and CO890 as ink diffusion at the boundary. Figure S14: digital photos of the PC paper swelled by Brij S2, Brij S10, and Brij S100. (b-c) SEM images of the surface of PC papers swelled by Brij S2 and Brij S10, respectively. The diameter of all the samples is 1 cm. Figure S15: (a) microscope images of the PC patterns fabricated with a half region swelled by CO720 and CO890 as inks of 0-24 h. (b) Diffusion distance of the inks as a function of time. (c) Reflection wavelengths of the G, Y, and R regions of patterns as a function of time and corresponding digital photos of the PC patterns. Figure S16: (a) microscope images of the PC patterns under 30, 50, 70, and 90°C of 0–60 min. (b) Diffusion distance of the temperatures as a function of time. The k1, k2, and k3 represent the slopes of 50, 70, and 90°C, respectively. Figure S17: multicolored micropatterns that can be repeatedly reconfigured. The scale bar can be used to the images in the same line. Figure S18: microscope images of the PC patterns at (a) pristine state, (b) 10 times, and (c) 20 times of being squeezed. Figure S19: reflection spectra of different samples under pressures. Figure S20: cross-sectional SEM images of the pattern “MPC” under different pressures. The scale bar is 100 *μ*m. Figure S21: digital photos of the MPC multicolor pattern that can be repeatedly reconfigured after being pressed. Figure S22: schematic illustration of two modes for the collection of the reflection spectra of the PC paper or PC patterns. Figure S23: schematic illustration of the details of testing the mechanochromic properties of the PC paper or PC patterns.
