# Supplementary material for: Reconfigurable Mechanochromic Patterns into Chameleon-Inspired Photonic Papers
Source: Research (Wash D C). 2022 Jul 19;2022:9838071. doi: 10.34133/2022/9838071 (PMC9343078; doi:10.34133/2022/9838071)
Supplement: Supplementary Materials — Figure S1: schematic illustration of two modes for the collection of the reflection spectra of the PC paper or PC patterns. Figure S2: schematic illustration of the details of testing the mechanochromic properties of the PC paper or PC patterns. Figure S3: optical properties and microstructure characterization of the PC paper. Figure S4: reflection spectra of the PC papers prepared with different volume fractions of silica particles. Figure S5: reflection spectra and corresponding digital photos of the PC papers self-assembled from silica particles with different sizes. Figure S6: SEM images of PC papers self-assembled by silica particles of different sizes. Figure S7: SEM images of the PC paper under (a) 0 and (b) 12 kPa. Figure S8: 3D reflection spectra of the PC paper with the pressure increased from 0 to 12 kPa. Figure S9: reflection spectra of the B, G, Y, and R points. Figure S10: mPC and mCO890 and the mCO890% and Δλ as a function of the thickness and fd of the PC paper. Figure S11: reflection spectra of the PC paper at a pristine state and in ethanol. Figure S12: reflection spectra of the PC paper at a pristine state and in water. Figure S13: CO720 and CO890 as ink diffusion at the boundary. Figure S14: digital photos of the PC paper swelled by Brij S2, Brij S10, and Brij S100. (b-c) SEM images of the surface of PC papers swelled by Brij S2 and Brij S10, respectively. The diameter of all the samples is 1 cm. Figure S15: (a) microscope images of the PC patterns fabricated with a half region swelled by CO720 and CO890 as inks of 0-24 h. (b) Diffusion distance of the inks as a function of time. (c) Reflection wavelengths of the G, Y, and R regions of patterns as a function of time and corresponding digital photos of the PC patterns. Figure S16: (a) microscope images of the PC patterns under 30, 50, 70, and 90°C of 0–60 min. (b) Diffusion distance of the temperatures as a function of time. The k1, k2, and k3 represent the slopes of 50, 70 [file 9838071.f1.docx]

**Reconfigurable mechano-chromic patterns into chameleon-inspired photonic papers**

Dongpeng Yang*^,1^, Yang Hu^1^, Dekun Ma^3^, Jianping Ge*^,2^, and Shaoming Huang*^,1^

^1^School of Materials and Energy, Guangzhou Key Laboratory of Low-Dimensional Materials and Energy Storage Devices, Guangdong University of Technology, Guangzhou 510006, P. R. China

^2^School of Chemistry and Molecular Engineering Shanghai Key Laboratory of Green Chemistry and Chemical Processes, East China Normal University, Shanghai 200062, China

^3^Zhejiang Key Laboratory of Alternative Technologies for Fine Chemicals Process, Shaoxing University, Shaoxing 312000, P. R. China

Correspondence should be addressed to Dongpeng Yang; [dpyang@gdut.edu.cn](mailto:dpyang@gdut.edu.cn), Jianping Ge; [jpge@chem.ecnu.edu.cn](mailto:jpge@chem.ecnu.edu.cn), and Shaoming Huang [smhuang@gdut.edu.cn](mailto:smhuang@gdut.edu.cn)


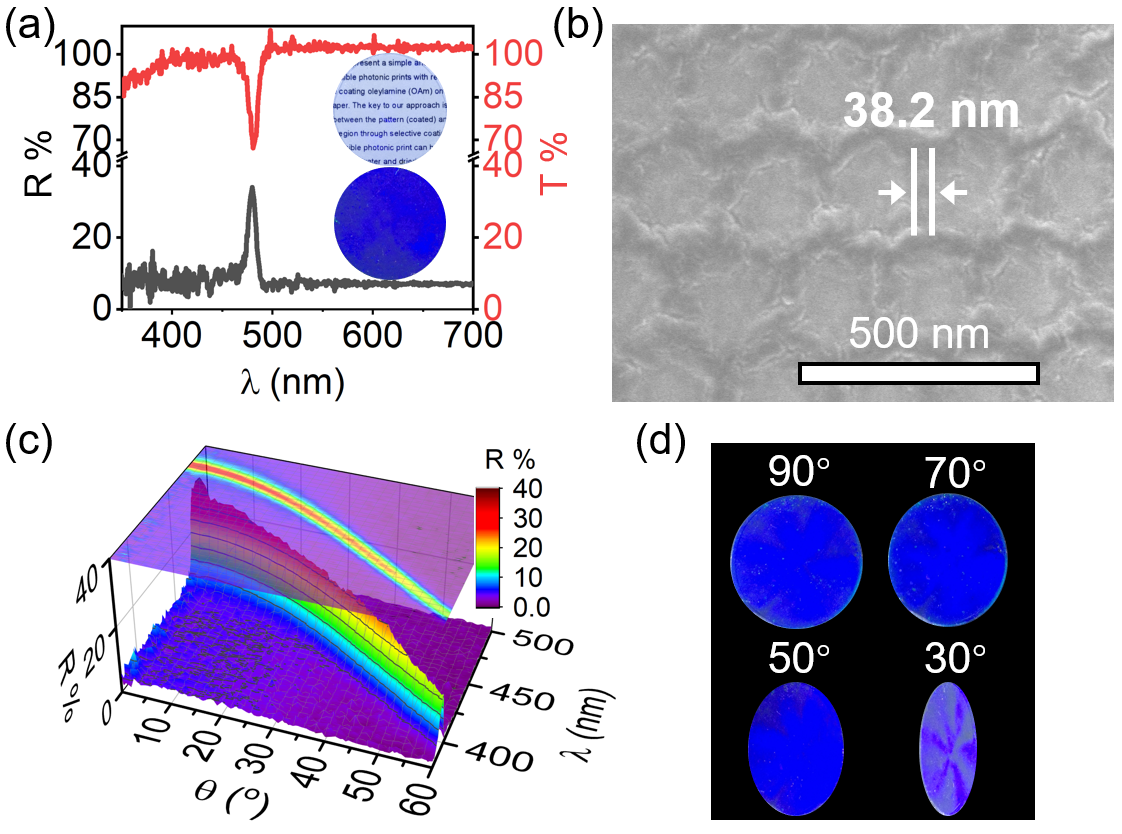


**Figure S1.** (a) Reflection and transmission spectra, and corresponding pictures of the PC paper. (b) SEM image of the non-closely packed structures of the PC paper. (c) Angle-resolved spectra, and (d) corresponding digital photos of the PC paper.


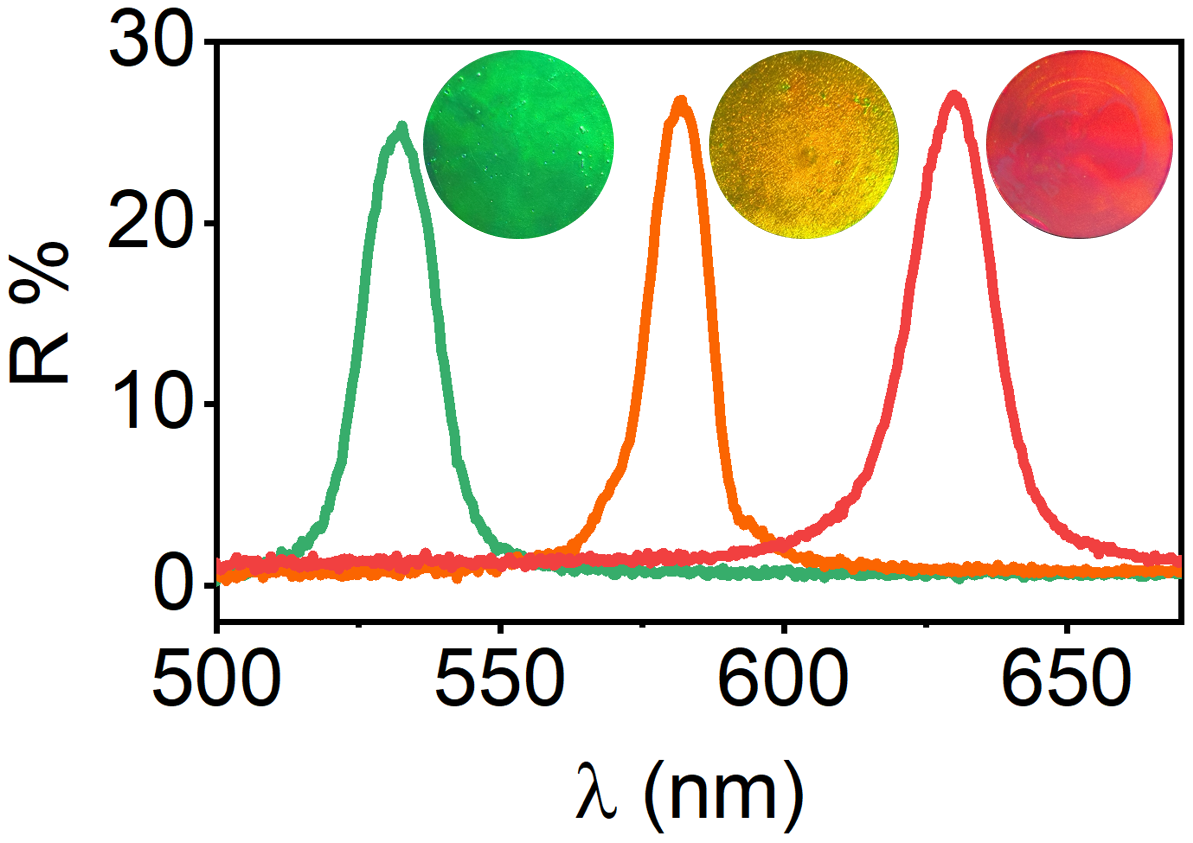


**Figure S2.** Reflection spectra and corresponding digital photos of the PC papers self-assembled from silica particles with different size. Particle size: 180, 192, and 213 nm (from left to right).


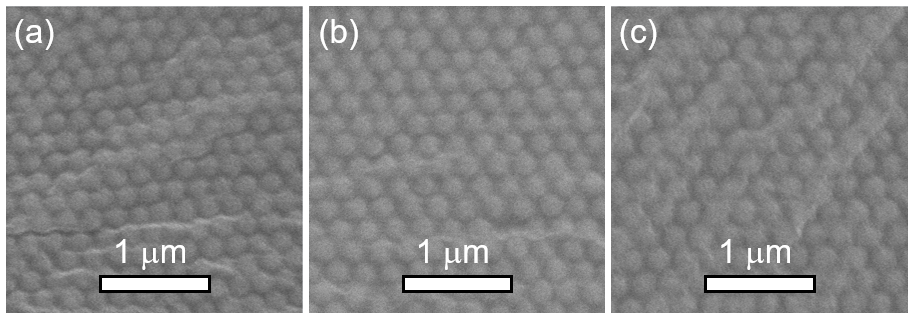


**Figure S3.** SEM images of PC papers self-assembled by silica particles of different sizes, (a) 180 nm, (b) 192 nm, and (c) 213 nm.


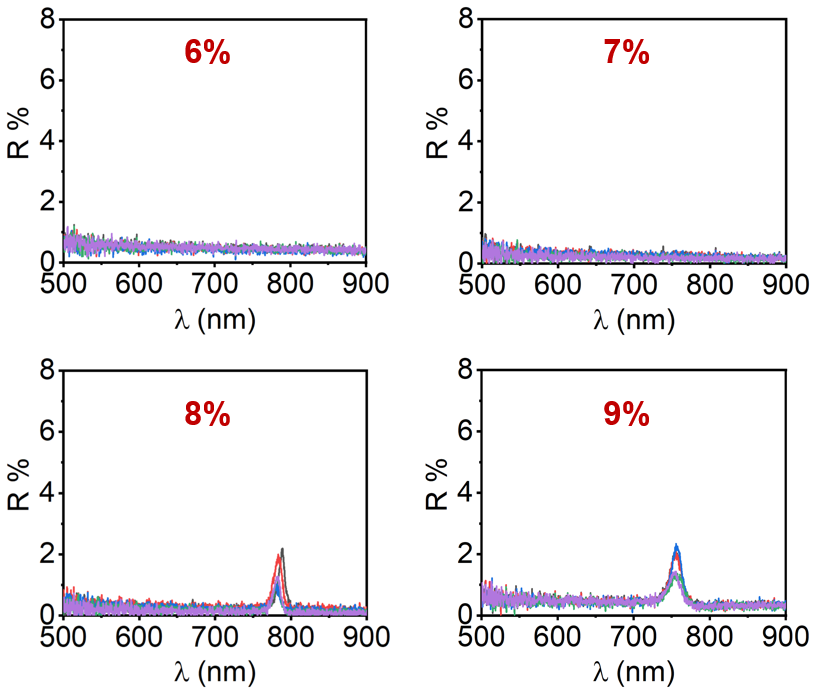


**Figure S4.** Reflection spectra of the PC papers prepared with different volume fraction of silica particles.


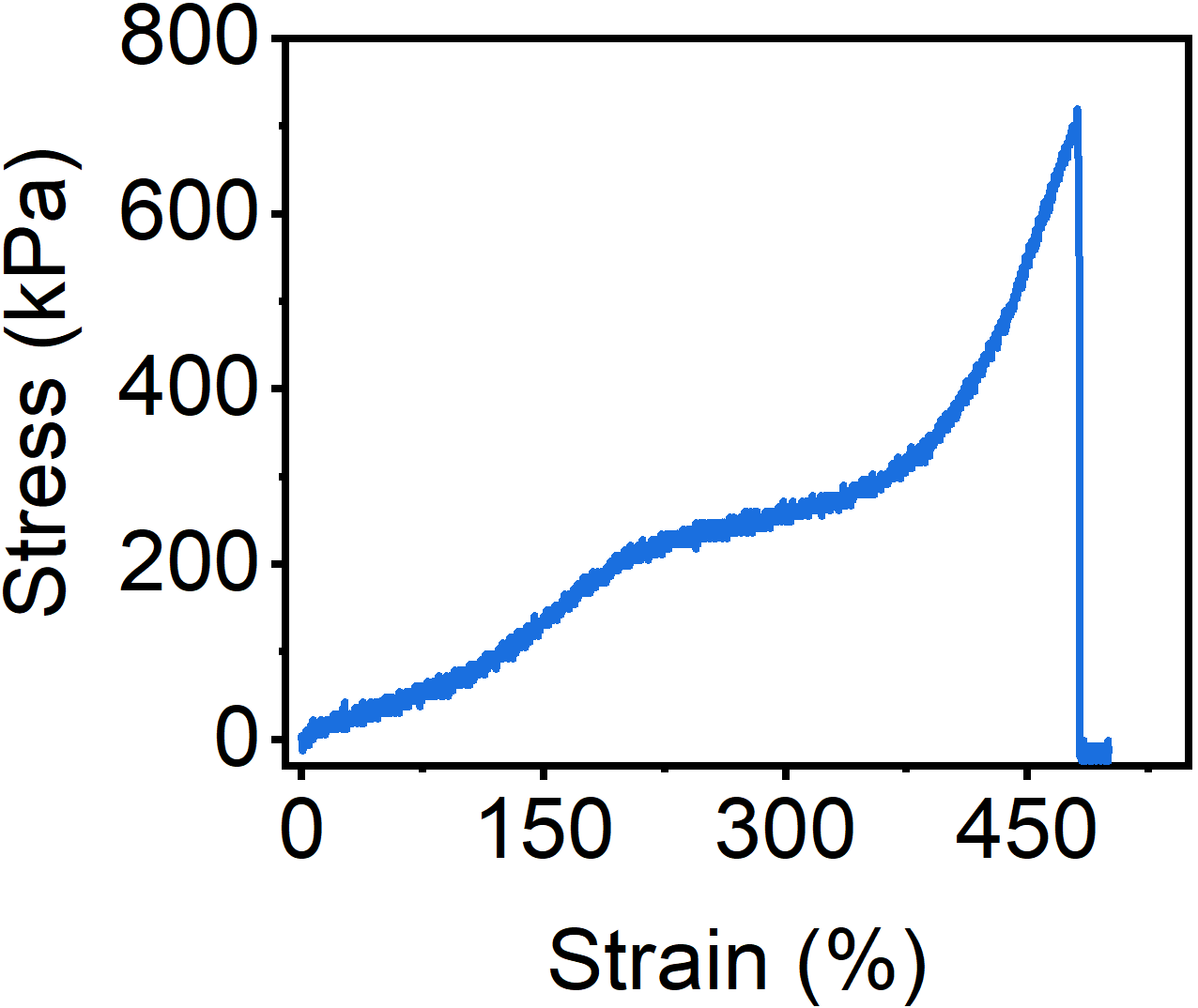


**Figure S5.** Tensile stress-strain curves of PC paper.


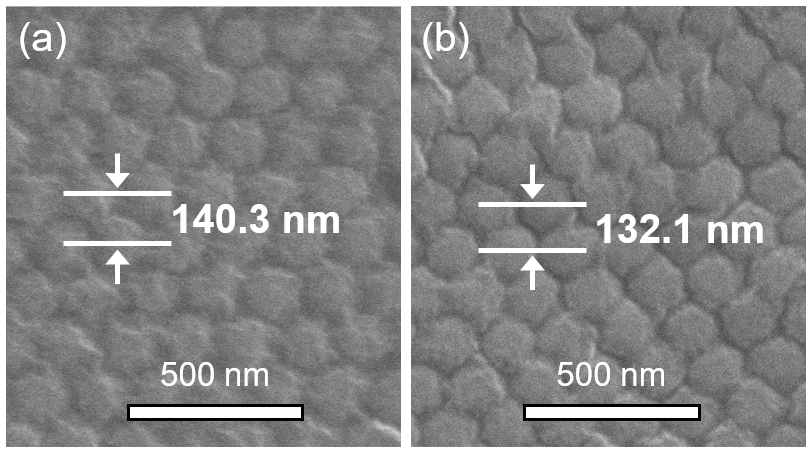


**Figure S6.** SEM images of PC paper under (a) 0 and (b) 12 kPa.


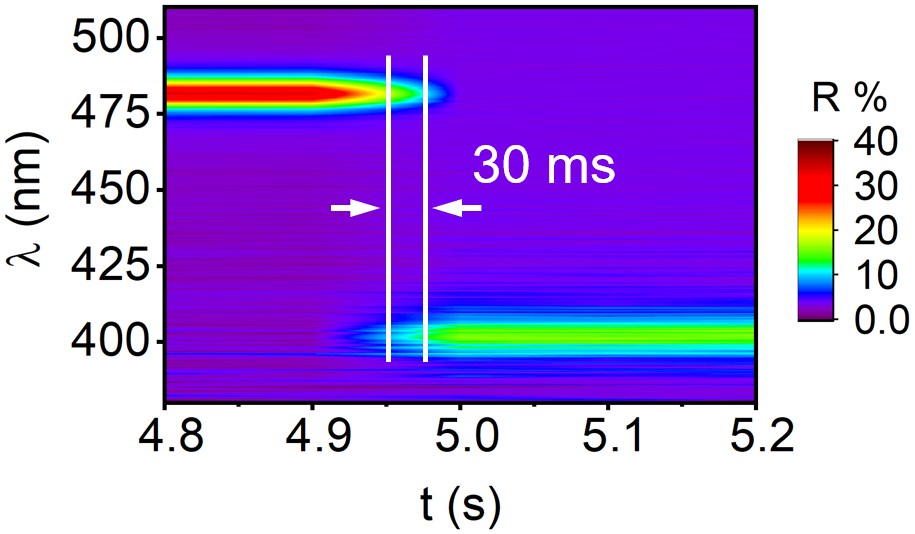


**Figure S7.** 3D reflection spectra showing the variation of reflection signal of PC paper with the pressure increased from 0 to 12 kPa.


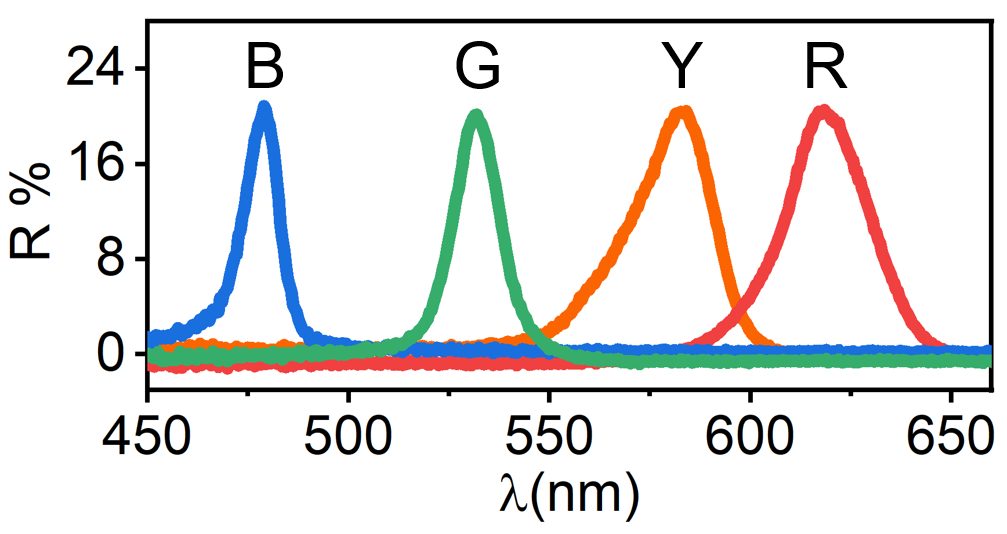


**Figure S8.** Reflection spectra of the B, G, Y, and R points.


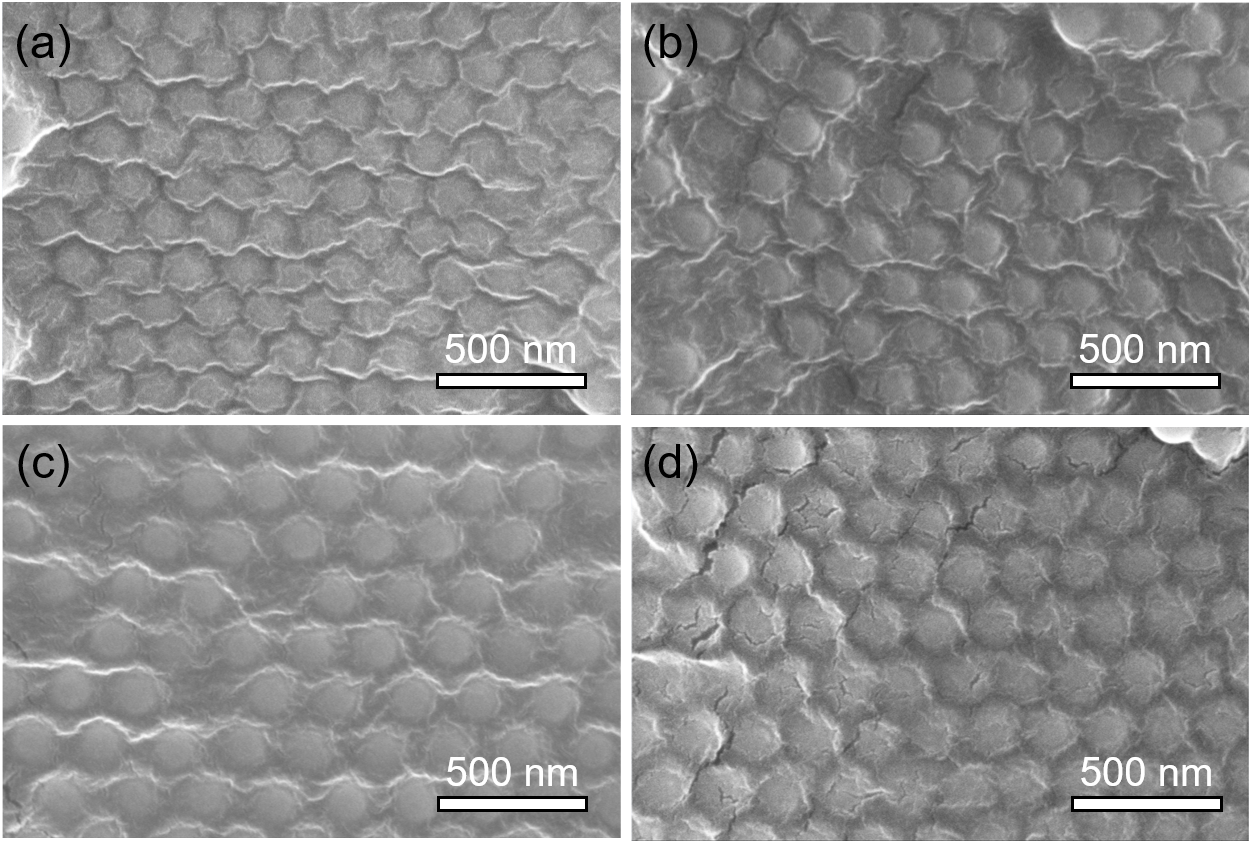


**Figure S9.** (a-d) Cross-sectional SEM images of patterned regions with blue, green, yellow, and red colors, respectively.

In addition to the swelling time, other parameters including the thickness of the PC paper and the f_d_ also have significant influences on the optical properties of the patterns. Since the Δλ is proportional to the m_CO890_%, both Δλ and m_CO890_% can be used to determine the optimized parameters of PC paper for the fabrication of PC patterns. The m_CO890_% can be calculated by eq 5, where m_CO890_ and m_PC_ are the mass weight of CO890 and PC paper, respectively.

m_CO890_% = m_CO890_/(m_CO890_ + m_PC_) = 1/(1 + m_PC_/m_CO890_) (5)

We firstly investigate the effect of thickness of the PC on the Δλ. Here, PC papers with the same reflection wavelengths located at 480 nm and f_d_ fixed to 60% but different thicknesses (90-270 μm) are firstly prepared. All the PC papers are swelled by CO890 under the same conditions with the swelling time fixed to 12 min. As shown in Figure S10a, the m_pc_ increases due to the increase of thickness of the PC paper, while the m_CO890_ is almost independent of the thickness. As the swelling occurs on one side of the PC paper, the amount of CO890 swelled into these PC papers depends on the f_d_. Therefore, the values of m_CO890_ of all these PC papers are almost identical since they have the same f_d_. As a result, m_CO890_% decreases dramatically from 24.7 to 9.5%, and the Δλ decreases from 101 to 35 nm (Figure S10b) when the thickness of the PC paper increases from 90 to 270 μm, demonstrating that a thinner PC paper will be favorable for the swelling. Apparently, a thin PC paper accompanied with a large m_CO890_% could possess a large lattice distance and thus a large Δλ. For practical usage, a large Δλ is always highly desired and required for multicolor prints. In this regard, PC paper with 90 μm should be a good choice for multicolor printing in this work.

In addition, the f_d_ also has significant effects on the Δλ of the patterns. Here, PC papers with reflection wavelengths located at 480 nm and thickness fixed to 90 μm but different f_d_ (60-85%) are prepared. For comparison, these PC papers are also swelled by CO890 with the swelling time fixed to 12 min. As presented in Figure S10c, the m_pc_ decreases as the f_d_ increases since the density of DEGEEA is much smaller than that of silica particles. In contrast, the m_CO890_ is proportional to the f_d_ because the PC paper with high f_d_ and low f_s_ can accelerate the swelling of CO890, resulting in the fast increase of lattice distance of PC paper and thus a large Δλ. Consequently, the m_CO890_% increases from 24.7 to 35.6%, and the corresponding Δλ increases from 101 to 182 nm (Figure S10d) when the f_d_ gradually increases from 15 to 40%. Therefore, PC paper with a high value of f_d_ will facilitate the swelling process and induce a large Δλ. Despite this, the swelling speed of PC paper with high f_d_ is too fast to precisely control the reflection wavelength of the patterned regions. According to our experiences, f_d_ of 60% is a suitable value because Δλ is large enough and can be precisely controlled through simply altering the swelling time.


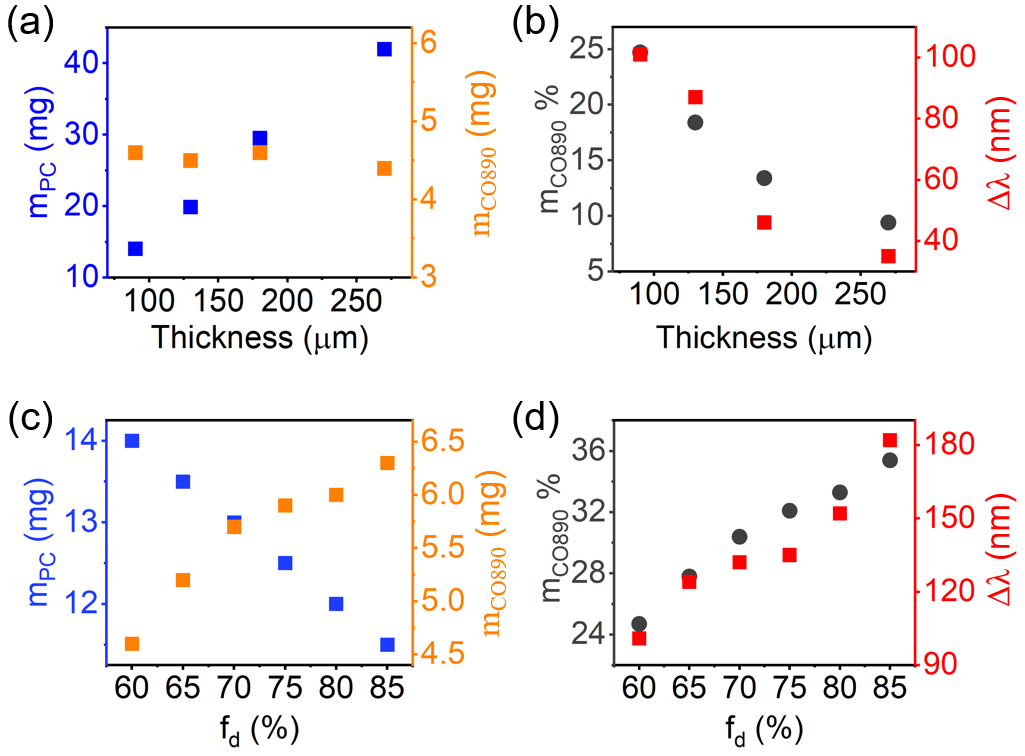


**Figure S10.** (a, c) m_pc_ and m_CO890_, and (b, d) the m_CO890_% and Δλ as a function of the (a, b) thickness and (c, d) f_d_ of PC paper.


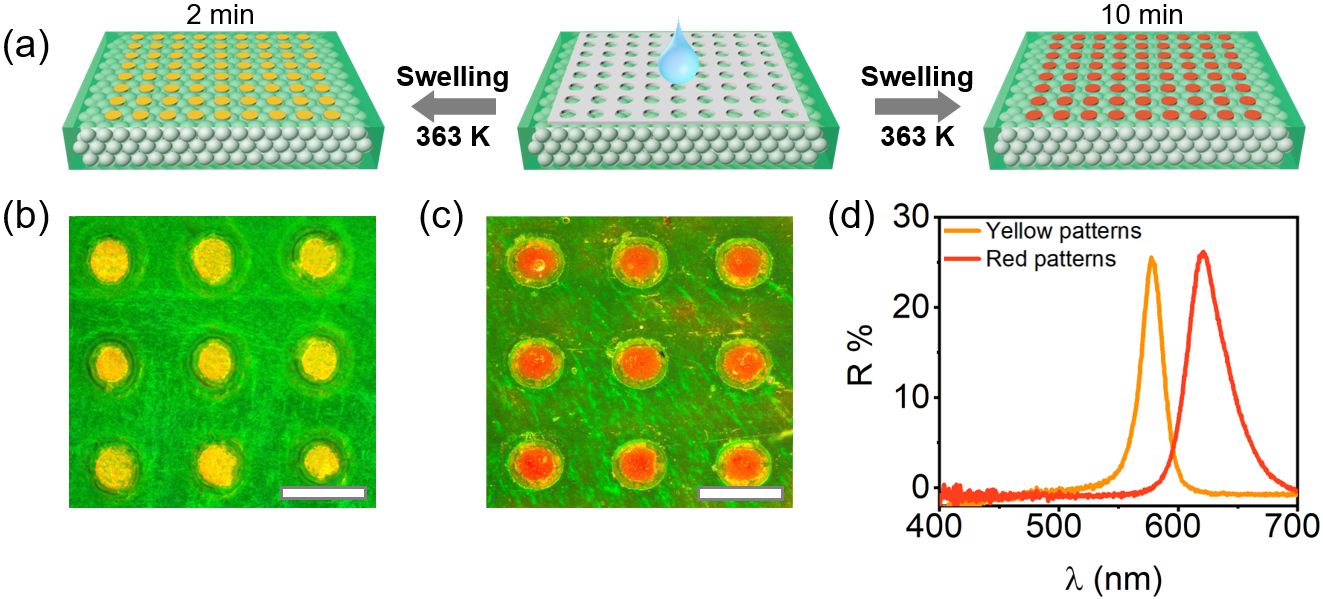


**Figure S11.** (a) Schematic illustration of the fabrication of high-resolution patterns. (b-c) Microscope images of yellow and red patterns, respectively. (d) The reflection spectra of the yellow and red patterns. The scale bar is 500 μm.


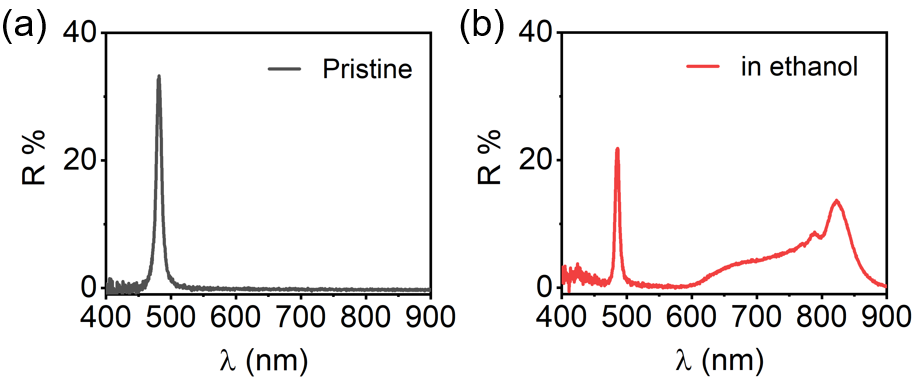


**Figure S12.** Reflection spectra of PC paper at (a) pristine state and (b) in ethanol.


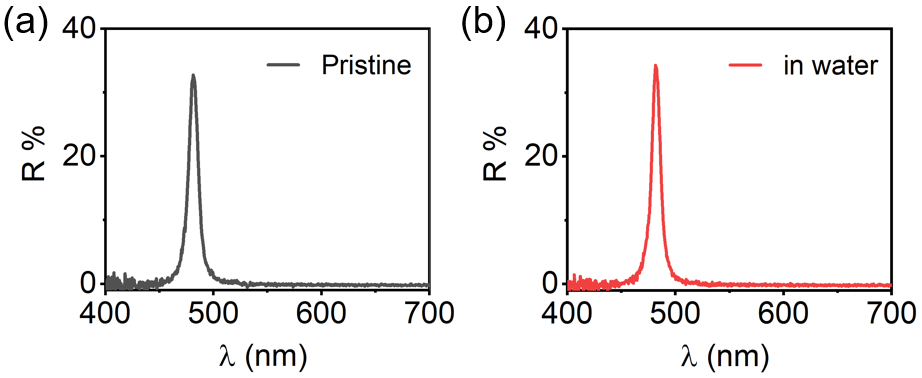


**Figure S13.** Reflection spectra of PC paper at (a) pristine state and (b) in water.


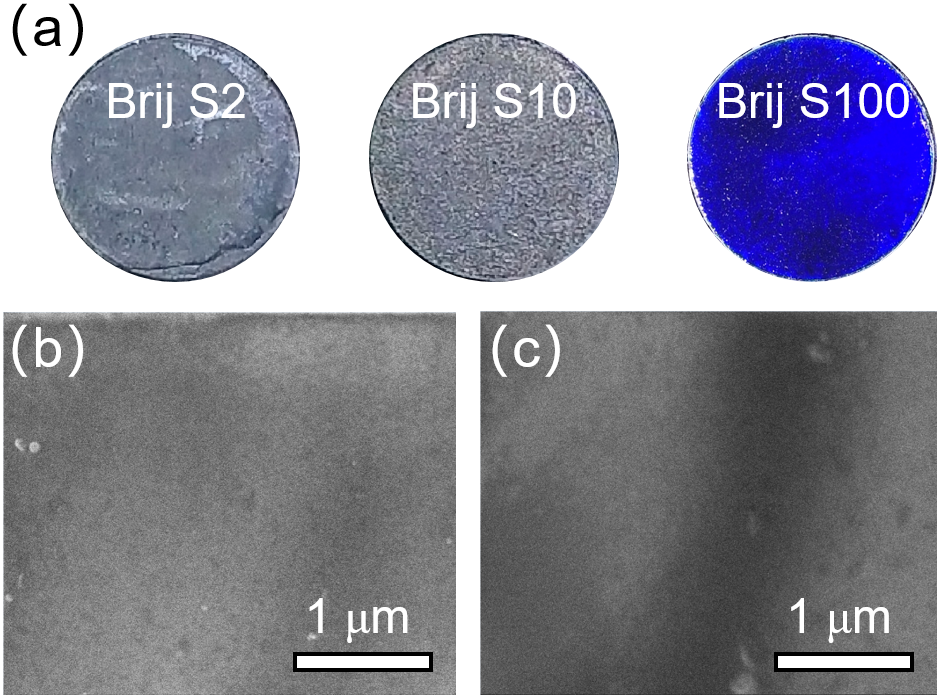


**Figure S14.** Digital photos of PC paper swelled by Brij S2, Brij S10, and Brij S100, respectively. (b-c) SEM images of the surface of PC papers swelled by Brij S2 and Brij S10, respectively. The diameter of all the samples is 1 cm.


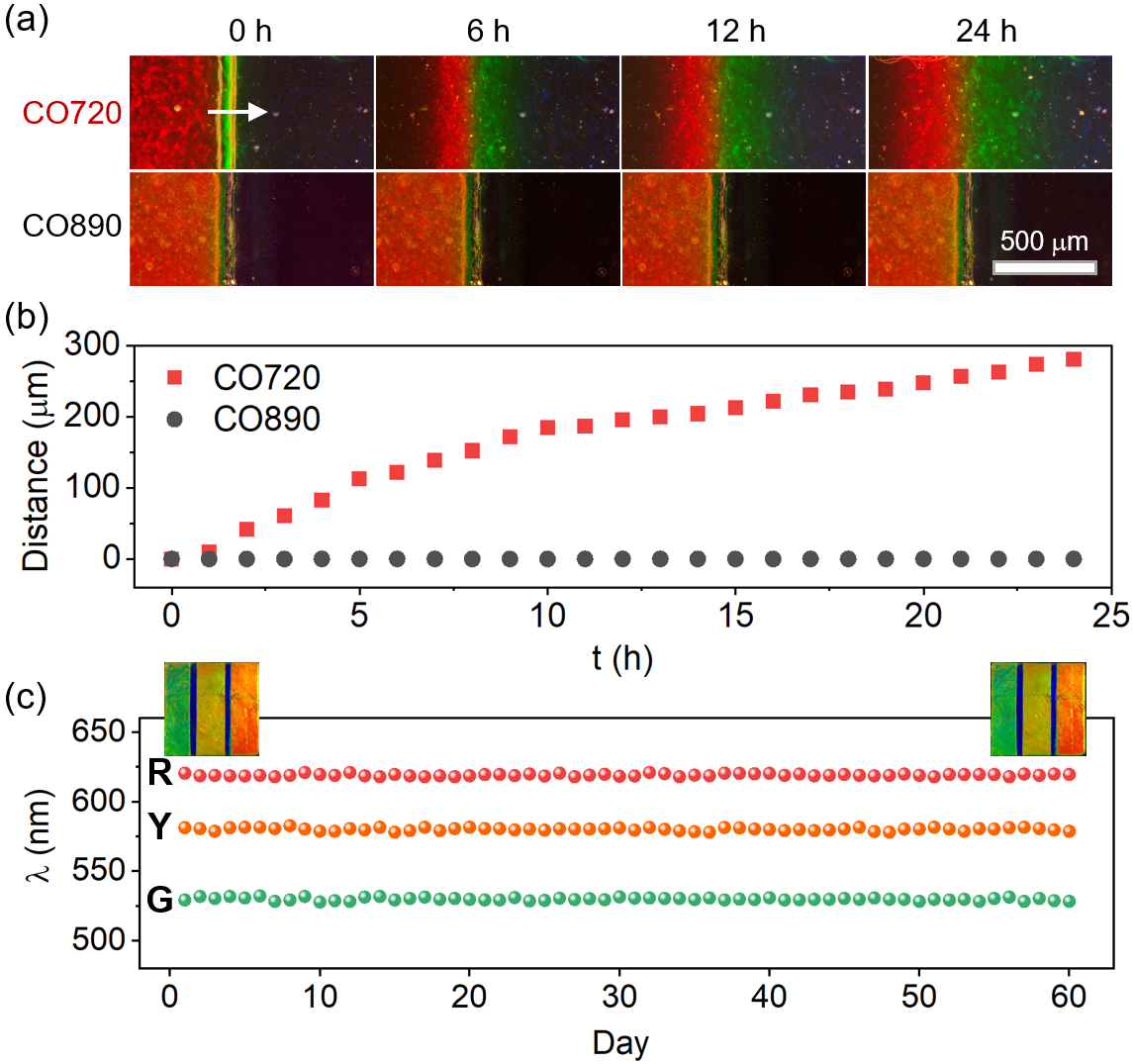


**Figure S15.** (a) Microscope images of the PC patterns fabricated with half region swelled by CO720 and CO890 as inks of 0-24 h. (b) Diffusion distance of the inks as a function of time. (c) Reflection wavelengths of the G, Y, and R regions of patterns as a function of time and corresponding digital photos of the PC patterns.


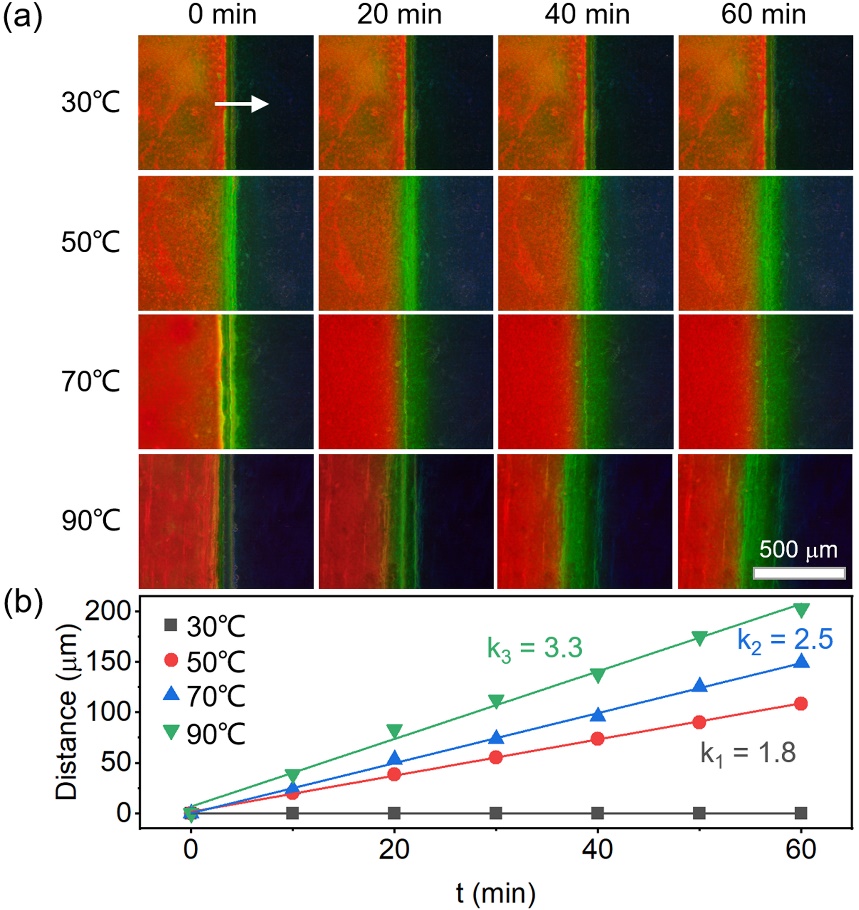


**Figure S16.** (a) Microscope images of the PC patterns under 30, 50, 70, and 90 ℃ of 0-60 min. (b) Diffusion distance of the temperatures as a function of time. The k_1_, k_2_, and k_3_ represent the slopes of 50, 70, and 90 ℃, respectively.


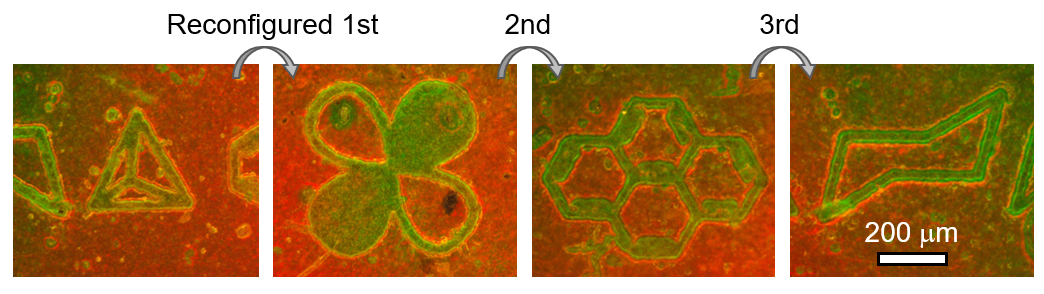


**Figure S17.** Multicolored micropatterns that can be repeatedly reconfigured. The scale bar can be used to the images in the same line.


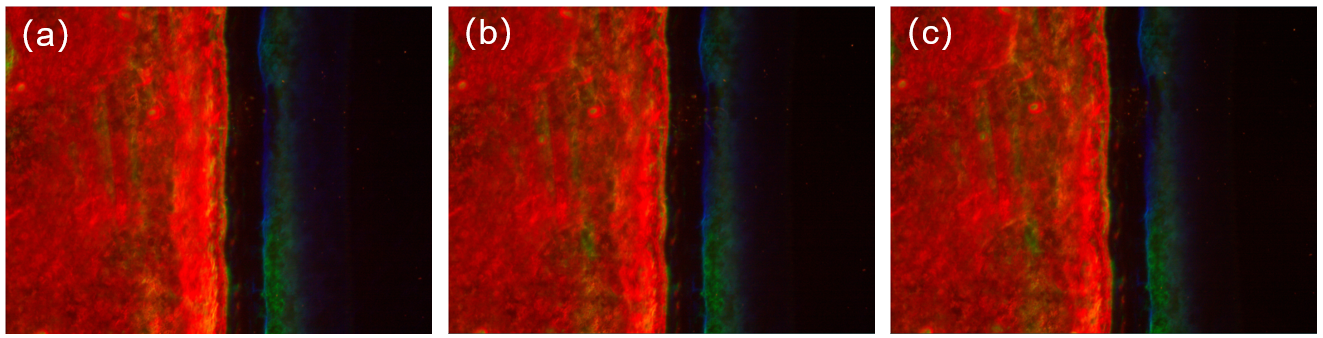


**Figure S18.** Microscope images of the PC patterns at (a) pristine state, (b) 10 times, and (c) 20 times squeeze.


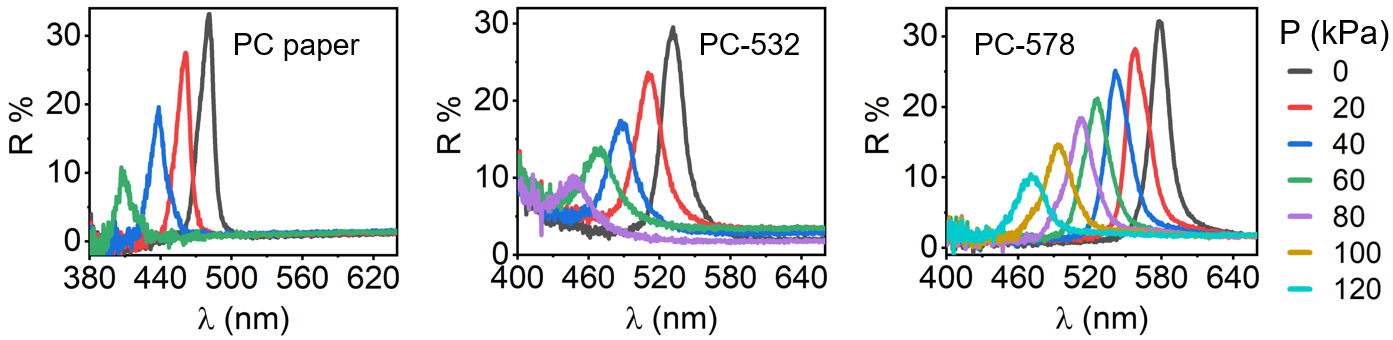


**Figure S19.** Reflection spectra of different samples under pressures.


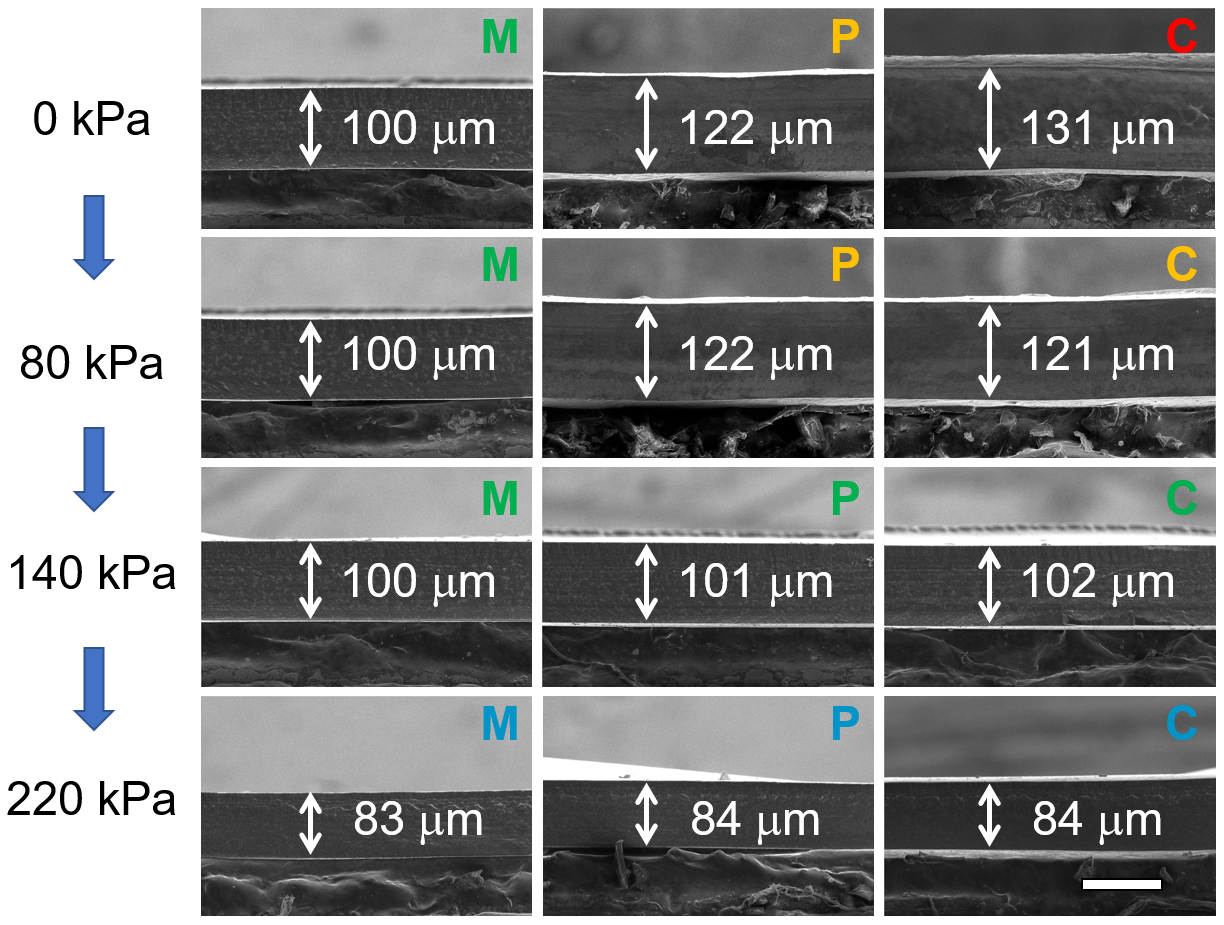


**Figure S20.** Cross-sectional SEM images of the pattern “MPC” under different pressures. The scale bar is 100 μm.


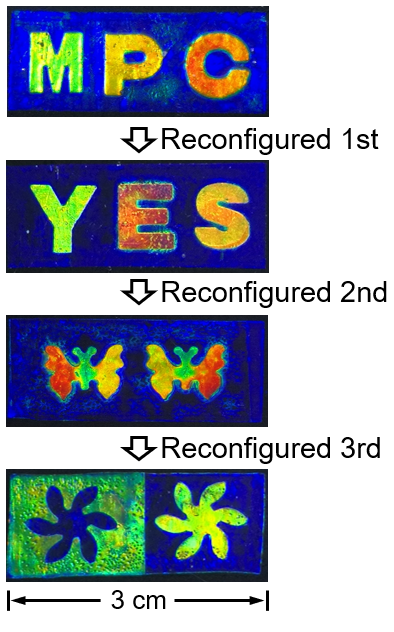


**Figure S21.** Digital photos of MPC multicolor pattern that can be repeatedly reconfigured after pressed.


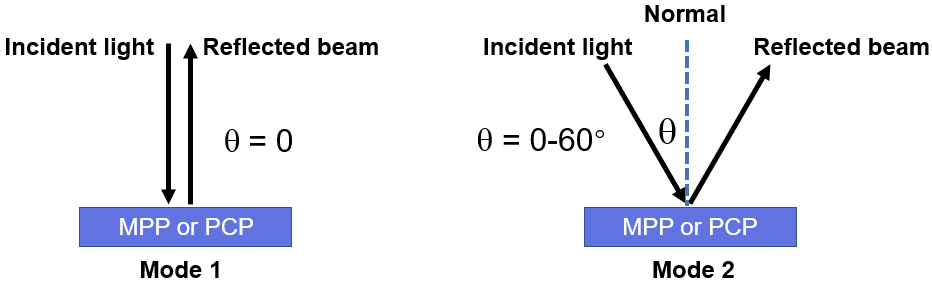


**Figure S22.** Schematic illustration of two modes for the collection of the reflection spectra of PC paper or PC patterns.


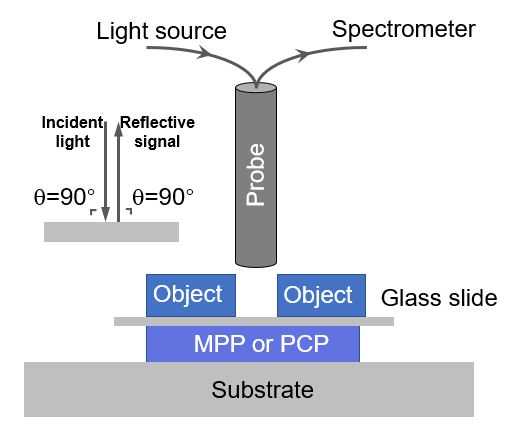


**Figure S23.** Schematic illustration of the details of testing the mechano-chromic properties of PC paper or PC patterns.
